# Life, its origin, and its distribution: a perspective from the Conway-Kochen Theorem and the Free Energy Principle

**DOI:** 10.1080/19420889.2025.2466017

**Published:** 2025-02-17

**Authors:** Chris Fields, Michael Levin

**Affiliations:** a Allen Discovery Center, Tufts University, Medford, MA, USA; b Wyss Institute for Biologically Inspired Engineering at Harvard University, Boston, MA, USA

**Keywords:** Diverse intelligence, drake equation, fermi paradox, free will theorem

## Abstract

We argue here that the Origin of Life (OOL) problem is not just a chemistry problem but is also, and primarily, a cognitive science problem. When interpreted through the lens of the Conway-Kochen theorem and the Free Energy Principle, contemporary physics characterizes all complex dynamical systems that persist through time as Bayesian agents. If all persistent systems are to some – perhaps only minimal – extent cognitive, are all persistent systems to some extent alive, or are living systems only a subset of cognitive systems? We argue that no bright line can be drawn, and we re-assess, from this perspective, the Fermi paradox and the Drake equation. We conclude that improving our abilities to recognize and communicate with diverse intelligences in diverse embodiments, whether based on familiar biochemistry or not, will either resolve or obviate the OOL problem.

## 1. Introduction

The Origin of Life (OOL) problem is ordinarily thought of as a chemistry problem [1]: how can the right molecules, under the right conditions, be compartmentalized in a way that initiates an autocatalytic process that maintains not just the right molecular concentrations, but also the enabling conditions and the requisite compartmentalization? Many ideas have been suggested, typically focused on either autocatalysis or compartmentalization as the primary problem. It is widely believed that multiple OOL events could have occurred on the early Earth, with only one lineage – that of the last universal common ancestor (LUCA) of all known life – surviving [2–4]. It is also widely believed that OOL events could occur elsewhere, prompting searches for evidence of extraterrestrial life by various means.

The OOL event leading to LUCA and its lineage is important in a number of related fields. First, it represents an originating singularity that must back-stop the reproductive cycle that is a cornerstone of evolutionary biology. Second, by effectively defining “synthetic” life, it sets a boundary condition on synthetic biology and bioengineering, both efforts to create and study life-as-it-could be [5] in novel implementations. Third, it is a critical fixed point for exo-biology, pushing us to define what it is that we are looking for on worlds beyond our planet, and to construct models of what different origin stories would mean for the further development of complexity and intelligence. Most crucially, it strongly impacts the very definition of “life”: a standard account in which life is a distinct category set apart from non-living matter is challenged by the need to identify and explain a sharp phase transition in which life arose from non-life. Related to this are questions of the relationship of life and mind. Whatever theories we formulate for the minimal dynamics needed to create minds, it is imperative to understand their relationship to the OOL. The conventional view is that cognition arose at some point during life’s expansion along an axis of complexity, i.e. that cognitive beings are a subset of living beings [6].

Here, we review an alternative way of thinking about the OOL problem that is motivated by the Conway-Kochen (CK) theorem [7,8], a result in fundamental physics, and the Free Energy Principle (FEP), a theoretical framework that originated in neuroscience [9,10] and has been extended over the past decade into a description of generic physical systems [11–16]. The CK theorem characterizes all physical systems as *agentic* in the specific sense of displaying behavior that is not, even in principle, predictable from local causal influences. The FEP characterizes all physical systems as *inferential* in the specific sense of behaving as Bayesian satisficers. Together, the CK theorem and the FEP suggest a view of cognition that is substantially broader than mainstream views in biology, e.g. as represented in [6], and may even surprise advocates of basal cognition in, e.g. plants or microbes [17–21]. They suggest, in particular, that all complex systems are organizations of *agential materials* that perform Bayesian satisficing on multiple scales [22–24]. This in turn suggests that the OOL problem is not, or at least not just, a chemistry problem, but is rather a cognitive science problem: the problem of characterizing the *level* or *type* of cognition at which it becomes meaningful for a system to be considered living. It raises the questions of whether life and cognition are co-extensive, as many have argued [25–28], and of what specific attributes distinguish living cognitive systems from non-living cognitive systems if they are not. It suggests that developing methods to both identify and communicate with diverse intelligences with diverse embodiments that exhibit behavior in diverse state spaces [22,29–31] will be required to fully address the OOL problem.

We first review the CK theorem in § 2 and the FEP in § 3. We then consider the fundamental question of whether life is co-extensive with cognition in § 4. We discuss the extraordinary range of possible embodiments, over essentially arbitrary spatial and temporal scales, that become available when the materials available in the universe are considered agential. We also consider the variety of state spaces that such systems may traverse over their “lifespans”, some of which may be only minimally coupled to our familiar three-dimensional (3d) embedding space in which the overt behavior of familiar organisms unfolds. We formulate typical criteria for life, including reproduction with variation and autopoietic metabolism, in ways that can be applied to diverse embodiments inhabiting diverse state spaces. We then turn in § 5 to Enrico Fermi’s famous “Where are they?” question, and discuss the kinds of communication capabilties we might expect diverse cognitive systems – which we may or may not consider to be “alive” – to be capable of employing.

## 2. The Conway-Kochen theorem as a generic characterization of agency

**Figure 1.**
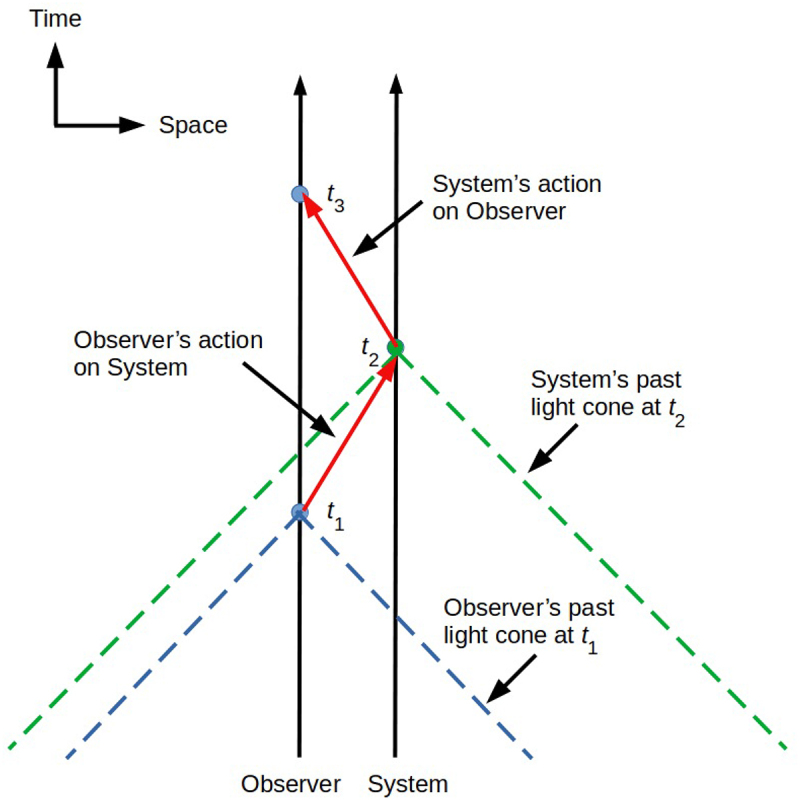
The setting for the CK theorem: an observer (left black vertical world line) interacts with a system of interest (right black vertical world line). Discrete exchanges of information are shown as red arrows; each exchange traverses space (horizontal axis) and takes time (vertical axis). The observer’s past light cone (blue dashed lines) at $t_{1}$ when action on the system is initiated (lower blue disk) lies entirely within the system’s past light cone (green dashed lines) at $t_{2}$ when the system receives the action (green disk).

In 2006, John Conway and Simon Kochen published their “free will theorem”; they published a more general, and hence stronger, version in 2009. The CK theorem concerns interactions between an observer and some system of interest that comply with both Special Relativity (SR) and Quantum Theory (QT). The former requires that interactions between system and observer are causal, in the specific sense of transferring information between systems i) forward in time and ii) at no more than the speed of light; indeed compliance with these two conditions define “causality” in SR. Figure 1 shows an interaction meeting these conditions. At every point on the “world line” depicting either system’s trajectory through spacetime, here for simplicity drawn vertically to indicate the passage of time with no (relative) motion in space, possible past causal influences on either system are contained with that system’s “past light cone”, which is the region of spacetime, extending arbitrarily into the past, from which a signal propagating at no more than the speed of light could reach the system. As shown in Figure 1, the past light cone of the system of interest at time $t_{2}$, when it receives an action by the observer, entirely contains the past light cone of the observer at $t_{1}$, when action on the system was initiated. Likewise, the past light cone of the observer at $t_{3}$, when it receives an observational outcome from the system, entirely contains the system’s past light cone at $t_{2}$.

The CK theorem in its original form states that in any observational situation compliant with SR and QT, if the actions of the observer at some time $t_{1}$ are not completely determined by the events in the observer’s past light cone at that time, then the observed system’s response, at some $t_{2}>t_{1}$ to the observer’s actions at $t_{1}$, are not completely determined by the events in the system’s past light cone at $t_{2}$. The proof is somewhat involved, and depends on the earlier Kochen-Specker theorem [32] showing that observations compliant with QT exhibit non-causal dependencies on context. The underlying intuition, however, is straightforward: the evolution of the quantum state of part of the universe can only be fully understood as a projection of the evolution of the quantum state of the entire universe. Local information, e.g. the information available from a past light cone, is never sufficient to completely specify a quantum state; this insufficiency of local information also underlies the Wootters-Zurek “no-cloning” theorem [33]. This is particularly clear in the case of spatially extended systems in entangled states, in which spacelike-separated components of a single system appear, in the laboratory reference frame of an observer, to have distinct past light cones. The insufficiency of local information for quantum state specification applies to both the observer and the observed system, replacing the “if – then” formulation of the original theorem with the stronger formulation of the 2009 version.

The CK theorem shows us that, at least in the context of current fundamental physics, *all* systems exhibit one of the typical hallmarks of living systems: their resistance to local, causal determination of behavior, and hence their ability to act in ways that are surprising. It is possible to write this off as “randomness” or “stochasticity” and hence to view it in essentially thermodynamic terms, but we note that doing so already provides the minimal requirement – a degree of autonomy – for a system to exist on the spectrum of agency, which can then be scaled up into higher forms of intelligence (with greater historicity, metacognition, etc.) by the processes of evolution or engineering. Here, we follow Wheeler [34], Fuchs [35], and others in interpreting the CK theorem, and indeed QT generally, as implying that all systems have a fundamentally agentic character. We will describe one way of formulating this in the next section: if two systems are distinguishable – formally, if their joint state is not entangled – they have independent and hence “free” choice of computations to perform on, and hence semantics to assign to, their inputs [36]. That such freedom of interpretation is the basis of agency is a key tenant of 2nd-order cybernetics [37–39].

## 3. The FEP as a generic characterization of cognition

The FEP describes the behavior of physical systems that can be distinguished from the environments in which they are embedded, and provides a criterion that such systems must satisfy in order to remain distinguishable from their environments over some period of time. It can be stated, in obviously tautological form, as the claim that any system that is distinguishable from its environment must behave in a way that maintains its distinguishability from its environment, from the perspective of an external observer, and possibly from its own perspective [11]. More technically, any such system must behave in a way that maintains the conditional statistical independence of its states from the states of its environment. The FEP describes the maintenance of conditional statistical independence as the asymptotic minimization of a variational free energy (VFE) [40] measured at the system – environment boundary, and describes the minimization of VFE as Bayesian satisficing. Intuitively, VFE is a measure of the system’s uncertainty about what its environment will do to it next; hence VFE can be interpreted as a measure of the stress imposed on the system by its environment, and Bayesian satisficing can be interpreted as deciding how best to relieve this stress. The FEP can thus be viewed as providing a dictionary for translating descriptions of behavior in the languages of either classical dynamical systems [11–14] or QT [15,16] into the languages of the cognitive, and when many agents are involved, the social sciences. We will, in what follows, consistently use the term “agent” to refer to a system compliant with both the CK theorem and the FEP, recognizing that the scope of this usage comprises all physical systems that remain distinct from their environments over some time period of interest, and that this scope is considerably broader than that of some other usages of this term; see [41] for a general review and [42] for a review specific to biology.

To see how the FEP works, let U (for “universe”) be some collection of physical degrees of freedom that is large enough to be considered isolated, and consider factorizations U=SE of U into a system S and its environment E, by definition everything other than S. Note here that the relevant state space for the FEP, the state space of U, comprises all possible states of U as a whole, not just the possible states of S. The FEP describes the behavior of the two factors, or components, S and E of U if and only if their states – which can be viewed as orthogonal projections of states of U – are conditionally statistically independent of each other. Conditional statistical independence of S from E and vice-versa requires i) that S and E are both sufficiently large components of U, i.e. that they are large enough to have “internal” or “bulk” states not directly affected by their interaction, and ii) that the interaction is sufficiently weak, i.e. that the coupling between their degrees of freedom is sufficiently sparse. Both classical and quantum physics provide precise criteria for meeting these conditions. Classical dynamical systems factor into two conditionally statistically independent components if and only if a Markov blanket (MB) – a “small” set of states through which all interactions flow – can be defined between them [43]; see [11] for a definition in a generic classical setting. Quantum systems factor into two conditionally statistically independent components if and only if the joint state can be factored into individual component states, i.e. if and only if $\left|U\rangle =\right|\mathrm{SE}\rangle =\left|S\rangle \right|E\rangle$ in Dirac’s notation. If this condition fails, S and E are, by definition, entangled. If this condition holds, a holographic screen can be defined through which all interactions flow [36]; this holographic screen plays the same role in the QT description that an MB plays in the classical description.

**Figure 2.**
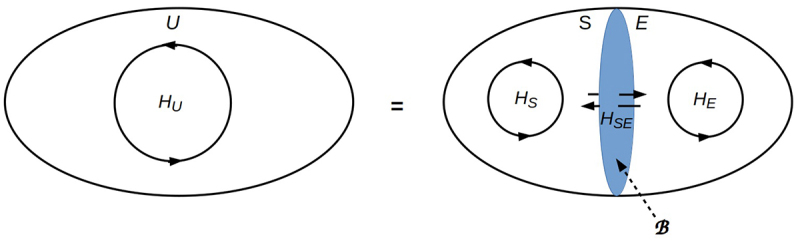
Decomposition of an isolated system U with internal dynamics $H_{U}$ into components S and E, with internal dynamics $H_{S}$ and $H_{E}$ respectively. The interaction $H_{\mathrm{SE}}$ between S and E is defined at the decompositional boundary B between them, and must be weak relative to $H_{S}$ and $H_{E}$ to maintain separability. Consistent with Figure 1, B has a temporal width set by the characteristic time of $H_{\mathrm{SE}}$; with a lower (i.e. high-energy) limit of twice the Planck time [36].

Given this description of S and E, the distinction between S and E persists through time if and only if the MB, or in the QT case, holographic screen, that defines their mutual boundary B persists through time. Note that this B is a boundary in the state space of U as shown in Figure 2, which may or may not correspond to a boundary in our familiar 3d embedding space. The boundary B is preserved, in turn, if and only if the interaction between S and E, which we will denote by the Hamiltonian operator $H_{\mathrm{SE}}$ that implements the interaction in QT, remains within bounds that i) permit sufficient thermodynamic free energy flows between S and E to power their respective internal processes [44], and ii) prevent any decrease in the number of “internal” states that constitute the respective “bulk” of S or E. The first of these conditions sets a lower bound on variation of $H_{\mathrm{SE}}$, while the second sets an upper bound. Such bounds on interactions are familiar in biology; they correspond to the bounds within which homeostasis (or allostasis or homeorhesis) can be maintained. If the size of the boundary B is allowed to vary slowly with respect to the “natural” timescale of $H_{\mathrm{SE}}$ while always maintaining homeostasis, these bounds correspond to bounds for allostasis or homeorhesis. Minimizing variation of $H_{\mathrm{SE}}$ within these bounds is minimizing VFE and hence stress. The FEP describes systems as generically preferring minimal stress, i.e. states that are as close as possible to optimal homeostasis.

The FEP is based on the recognition that the internal processes implemented by S and E – which we will denote by the Hamiltonian operators $H_{S}$ and $H_{E}$, respectively, that implement these processes in QT – can be viewed as representations, or in statistical language, generative models, of the interaction $H_{\mathrm{SE}}$. Each of S and E can, in this case, be regarded as continually predicting, by executing their internal processes $H_{S}$ and $H_{E}$, respectively, that their mutual interaction $H_{\mathrm{SE}}$ will remain within its homeostatic bounds, which are the bounds within which S and E remain separable and the internal processes $H_{S}$ and $H_{E}$ remain well defined. This mutual dependence between $H_{S}$, $H_{E}$, and $H_{\mathrm{SE}}$ is particularly clear in the QT representation, where by definition, the internal process of the total system U is $H_{U}=H_{S}+H_{E}+H_{\mathrm{SE}}$, and since U is by definition isolated, we can set $H_{U}=0$. We then have $H_{S}+H_{E}=-H_{\mathrm{SE}}$, so that if $H_{S}$ and $H_{E}$ remain within bounds required to maintain separability, $H_{\mathrm{SE}}$ must remain within a corresponding bound. Intuitively, if the dynamics of the internal bulks of a system and its environment are bounded, then their exchange must also be bounded. In this bounded condition, in which the bulk dynamics are individually well-defined and so can be viewed as implementing well-defined functions, S can be viewed as predicting the influence of E on its states, and vice versa [11].

For any system that implements a generative model of its interaction with its environment – effectively, a generative model of its expected inputs and available outputs – departures from model expectations can be represented by a variational approximation, namely a VFE [40]; again see [11] for a formulation in the current setting. The maintenance of $H_{\mathrm{SE}}$ within homeostatic bounds by the actions of $H_{S}$ and $H_{E}$ can, therefore, be represented as minimization of this VFE. Minimization of VFE over inputs can, however, be represented formally as Bayesian satisficing; hence the actions of $H_{S}$ and $H_{E}$ can be viewed as Bayesian satisficing, and the systems S and E can be viewed as Bayesian agents. In FEP language, S and E engage, at all times, in *active inference*, both adjusting their generative models to account for incoming data and acting on each other to maintain their incoming data within model-expected bounds, i.e. within the bounds required to maintain separability [45–47].

Treating physical interactions between separable agents as active inference, with the outputs (“actions”) of one being the inputs (“sensations”) of the other, redescribes such interactions as communication. How each party responds to communicative interactions depends on its internal process, i.e. on $H_{S}$ and $H_{E}$, respectively, for systems S and E. These processes generate actions from sensations, and hence encode, typically implicitly, the “differences that make a difference” to each system, and hence the semantics, in Bateson’s [48] sense, that they assign to their sensations [49,50]. As $H_{S}$ and $H_{E}$ are conditionally independent in separable systems, these semantics can be regarded as “freely chosen” in the sense of not locally determined, as employed in the CK theorem. Asymptotically, the FEP drives systems toward mutual predictability and hence a shared language, with generalized synchrony (i.e. synchronization of chaos) and entanglement as the asymptotic limits in classical and quantum treatments, respectively [15].

## Life = Cognition, or Life ⊂ Cognition?

4.

By recasting fundamental physics in agentive language in a way that applies to all time-persistent systems, the CK theorem and the FEP erase any “bright lines” separating cognitive systems from the “merely physical” while providing a natural association between dynamic and cognitive complexity. Relatively simple, intuitively “dumb” systems like electrons or rocks are negligibly cognitive, complex, multicomponent systems like macromolecules, biochemical pathways, or cells are significantly cognitive, exhibiting features such as error correction, learning, and memory [22], while even more complex systems like octopi, humans, or ecosystems are even more cognitive; in all cases, to a kind and degree that must be determind by experiment [51]. Indeed without further limiting assumptions, the CK theorem and the FEP are consistent with the radical panpsychism proposed by philosophers such as Galen Strawson [52] or Philip Goff [53]; see [54] for a cross-cultural historical review and [22,50] for specific discussions. However, since neither the CK theorem nor the FEP are themselves theories of consciousness *per se*, they by no means require it (Fields, Albarracin, Friston, Kiefer, Ramstead, and Safron, in review).

Erasing the line between the “merely physical” and the cognitive immediately raises the question of where life fits in. Are we justified in identifying the living with the cognitive, as argued by Maturana and Varela [25] or Pattee [26], if doing so extends the meaning of “living system” to include everything that persists through time? How important are often-cited functions such as replication, metabolism, or compartmentalization to the definition of “life” [55]? How important is organic chemistry – is it the exclusive source of complex systems, or can other chemistries also generate complex systems autonomously? Is it even worth trying to define life in general terms, given just the single example of the lineage of LUCA [56]? It is worthwhile to consider the use of terminology in asking these questions. It is often held that applying cognitive terms to unconventional substrates (biochemical pathways, cells, organs, “machines”, etc.) is useless wordplay at best, and a category error at worst. We propose an instrumentalist stance, where ontological categories are not taken as inviolable philosophical givens, but instead must be updated with the progress of science, and the degree of utility of terms comes from the interaction protocols they enable or suppress. For example, the statement that a system is roughly in position X on the spectrum of agency cashes out via the degree of empirical benefits provided by using tools from cognitive and behavioral science on that system. We have covered elsewhere [57–61] numerous examples of how such erasure of boundaries facilitates novel research programs and leads to new practical capabilities and discoveries, for example, in biomedicine and bioengineering. Thus, the idea that cognition is extremely widespread does not devalue the concept, but instead suggests experimental testing, in new systems, of tools and concepts that have proven useful for understanding conventional embodied minds

The FEP originated as a theory of living systems, and it generates many identified features of life from its minimal physical assumptions. All separable systems, for example, must be compartmentalized, although as noted, they may be compartmentalized in a space other than our 3d embedding space. Gene regulatory networks, for example, are compartmentalized in a state space of molecular concentrations, while execution traces of computer programs are compartmentalized in state spaces of possible encodings on finite data structures. As noted above, the FEP generically reproduces the preferences of living systems for low environment-induced stress. The FEP generically reproduces three of the four “pillars” advanced as definitive of life (or in their terminology, “lyfe”) by Bartlett and Wong [55], dissipation, homeostasis, and learning, though some systems display these functions only in some minimal-complexity limit. The fourth pillar, autocatalytic reproduction, is not required by the FEP; however, any persistent, bounded system that interacts with its environment must, by construction, be autocatalysic. In particular, the dynamics of an open system that is both bounded and persistent must have both dissipative and non-dissipative components that, in classical formulations, correspond to nonequilibrium steady-state solutions that break detailed balance. Systems in which dissipative fluctuations average to zero appear conservative, i.e. behave as classical objects, while systems in which dissipative dynamics remain significant are chaotic, exhibiting familiar characteristics of life, such as electrophysiological oscillations, biorhythms, lifecycles and reproduction [13]. Reproduction is, moreover, a natural strategy for any system attempting to reduce its local VFE, as putting a copy of yourself into your environment typically increases its predictability [62]. The FEP generically induces systems of sufficient complexity to develop neuromorphic morphologies to maximize information gain from their environments [63]. It generically induces compartmentalization and hierarchical information processing [14,64–66]. It induces communication and collective problem solving in groups of similar agents exposed to a shared environment, and hence provides natural descriptions of multicellular development, swarm behavior, and evolution at the population scale [67–70].

An obvious riposte to the claim that the FEP generically describes life is to point to complex artifacts with known structures, such as human-built computers, and to claim that they are obviously not alive. While such systems are compartmentalized, dissipative, and capable of learning, this argument goes, they cannot be alive because they do not maintain homeostasis and are not autocatalytic. We must ask, however, whether this is merely an intuition pump. Organic-chemistry based systems exist to which we supply a designer-perfect environment, arbitrarily-large supplies of thermodynamic free energy, and assisted kinematic replication with externally-supplied parts; examples include genomic DNA, proteins, and viruses. If we constructed computers from organic parts and maintained them inside our bodies, would we still regard them as non-living artifacts? Many organisms, including not only obligate human parasites and pathogens but also most domestic animals, rely on human-supplied environmental services to maintain homeostasis and reproduce – does this dependence on us render them any less alive? We ourselves are dependent on our microbial endosymbionts, as they are on us. All organisms are reliant on their environments; this lack of independence from the environment could be considered definitional. Historically, “we know it when we see it” has not been a reliable approach to life or mind. We would argue that it is even less so now [22,31].

If the FEP does generically describe life, where does this leave the OOL problem? Was there just one origin of life, at the Big Bang? Or are OOL events ubiquitous, occurring wherever organic or any other chemistry is available to generate complexity? If the FEP does not generically describe life, i.e. if living systems are some proper subset of agentive, cognitive systems, what must be added to the properties and capabilities that the FEP does render generic to produce a definition of life, and how can such additions be squared with the increasingly blurry lines between naturally-evolved systems and bio-engineered constructs [22,31]? Is, in other words, “life” an ontological category, or an interpretative construct [71]? One could, following [56], argue that such questions do not and even cannot matter. What clearly *does* matter is whether we can identify and communicate with the diverse intelligent systems that the FEP tells us will be ubiquitous, whenever we come across them.

## 5. Where are they?

The Fermi Paradox – why we have never observed evidence of intelligent extraterrestrial life even though the observable universe is large enough that we might expect to – has been widely discussed since [72]. Webb [73] reviews 75 proposed solutions, while Gray [74] advances a general critique of the discussion. As Webb points out, however, both the statement of the paradox and its proposed solutions implicitly model extraterrestrial life as similar to human life, psychologically and culturally even if not biologically [75]. This assumption adds drama, and lends much of the discussion an air of science fiction. The FEP models all systems as having a psychology that minimizes VFE, and hence experienced stress, a psychology that even individual cells can have [26]. It associates psychological complexity with system complexity, provided the bandwidth of the system’s boundary permits it [66]. Additional assumptions would, however, be necessary to predict a psychology similar to that of humans; hence the FEP tells us nothing about how prevalent a human-like psychology, let alone culture, might be. While we do not, moreover, have access to truly alien life, we do now have the opportunity to study synthetic and hybrid beings that have not been subject to the typical evolutionary selective forces across the tree of life. Cyborgs, hybrots, biobots, minimal active matter, and AI all present a unique challenge to the task of being able to predict emergent form and function when environment and heredity do not tell the whole story [23,76].

The FEP does, however, tell us that a cognitive system much more complex and intelligent that we are exists here on Earth. Phylogenetic evolution and individual morphogenetic development have the same formal structure when described by the FEP, differing only in spatial and temporal scale [77]; the entire lineage of LUCA can, therefore, be described as the morphogenesis of a single organism, Life as we know it [78]. We can characterize this organism genetically [4,79], and describe how its development has altered its environment [80], but our communications even with parts of it to which we are closely related are clumsy, and we have essentially no understanding, beyond the abstraction of VFE minimization and hence stress reduction, of its overall psychology. We are not equipped to “see” the environment of all of Life as we know it, and we cannot tractably model its overall interaction with its environment in any detail. Expanding the system of interest to the planetary Gaia of Lovelock and Margulis [80] – see [81] for a exobiological generalization – yields an even more complex system, some processes in which have been modeled as active inference [82], but to which the above comments about full or detailed models apply. What would it be like to communicate with this system? Can tools be developed to assist the mapping from theoretically predicted perceptual and goal spaces of systems such as these to our own – a kind of augmented reality to enable the understanding or even co-existence of highly diverse minds? Can a partial lifting of our innate mind-blindness be driven in the same way that a good theory of electromagnetism and its resulting technology enabled us to operate across an EM spectrum of whose existence we were not even aware? We do not know. The difficulties we have in recognizing perception-action loops, problem-solving behavior, and intelligent navigation of unconventional spaces of even our own natural organs [30] underscore the narrow filters that our evolutionary firmware provides.

A model of a physical system as an information processor – a computer – is a mapping from observable physics to a computable function [83]. The FEP is one way of specifying the general form of such mappings. Maps from observable physics to computable functions, including maps of the form specified by the FEP, can be constructed at any scale, with computational complexity decreasing as the scale over which events are coarse-grained increases. Within a given scale, many distinct maps are possible. Any physical system can, therefore, be viewed as simultaneously computing many distinct functions, with which function it is “seen” as computing depending on the perspective taken and the measurements made [84]. The FEP, therefore, not only describes generic systems as VFE minimizers at every scale [14]; it allows multiple such descriptions. Whether a description supports productive interactions with the system depends on how well it captures the way that the system itself measures and acts on its world – the “reference frames” that it employs to make sense of what its world is doing to it [15]. To understand, and especially to influence, how systems behave, it works best to communicate with them in their own languages. This is the phenomenon of scale- and description-dependent “causal emergence” [85], which we grasp intuitively with other people and, to some extent, with other mammals, can approach experimentally in the case of model organisms [86,87], but have only a theoretical grasp on in the case of generic systems.

The FEP thus suggests that the solution to the Fermi Paradox is to drop our anthropomorphism about intelligent life; see [88] for a similar conclusion from different reasoning. It suggests that we are surrounded by intelligent life, some much more intelligent than we are, that we cannot yet detect, or do not understand and cannot yet communicate with. It suggests that intelligent life pervades the universe, but that we should not expect it to resemble us in structure, embodiment, or habits. Supposing that we will encounter technological artifacts of a kind we might imagine someday building, or receive signals of a kind we might imagine someday sending, is supposing that intelligence implies a psychology, and a manipulative body, functionally similar to ours. The FEP suggests that life, and psychology, are far more diverse even than the lineage of LUCA, let alone the sublineage leading to mammals, and to us.

Even given such expectations, however, Earth-bound life provides sufficient diversity to challenge our abilities to construct models and conduct experiments. As [6] reveals, only a minority of life scientists accept that organisms are in general intelligent. We do not yet understand the sensory and behavioral capabilities of microbes in sufficient detail to adequately predict behavior in novel environments; our understanding of eukaryotic cells, e.g. human cancer cells, is even more limited. Comparative genomics continues to reveal novel, apparently lineage-restricted functions, and genomic sequence diversity is large enough that lateral contributions from non-LUCA lineages, as illustrated schematically in [4], cannot be ruled out. Synthetic biology and biorobotics are, moreover, now constructing living systems that have both no evolutionary histories in their current embodiments and apparently novel problem-solving capabilities [89–91]. On the more macroscopic side, ecology remains largely a science of observations and phenomenological models, and we have little appreciation of even eusocial insect colonies as cognitive individuals. We struggle with finding ways to communicate with collective intelligences, such as ant colonies (vs. the individual ants) [68], and are rarely aware that we ourselves are likewise examples of collective intelligence. We find it hard to recognize our cognitive kin at unfamiliar spatio-temporal scales or operating in unfamiliar problem spaces, and many scientists seem to work hard to maintain all manner of distinctions as a bulwark against losing what is left of anthropocentric ideas. Even the most basic aspect of being a real agent – having physical embodiment – is not nearly as solid as it once was. We can now understand ourselves as temporarily persistent, dynamical patterns – not objects – like solitons, hurricanes, etc. Our nature as self-modifying patterns within metabolic [92] and cognitive [93] media forces us to confront the question of what other patterns are actually agents? The tenuous distinction between patterns and objects – thoughts and thinkers – opens roads to recognizing an increasingly wide class of unconventional agents to which we had been completely blind [71]. We are, in other words, still only minimally prepared to confront the other living systems present on our own planet. Intelligent life originating on other planets, or its artifacts, may be unrecognizable by us.

## 6. The Drake Equation in light of the FEP

The Drake Equation estimates the number N of extraterrestrial intelligent systems detectable from Earth as $N=Rf_{p}n_{e}f_{l}f_{i}f_{c}L$, where R is the rate of star formation, $f_{p}$ is the fraction of stars with planets, $n_{e}$ is the number of life-supporting planets per such star, $f_{l},f_{i},$ and $f_{c}$ are the fractions of such planets that develop life, intelligent life, and technological cultures respectively, and L is the average time a technological culture is detectable [73]. The uncertainties in all of the factors except R and $f_{p}$ are very large, and published estimates of N vary by many orders of magnitude. Implicit in the Drake Equation is the assumption that detectable extraterrestrial intelligent systems have undergone evolutionary, cultural, and technological developments comparable to ours; such systems are, for example, often assumed to be intentionally broadcasting information about themselves or to be actively engaged in colonizing planets other than their own. Hence the Drake Equation is primarily concerned with the detection of artifacts – EM signals, material objects, or other information-bearing patterns that cannot be attributed to “natural causes” given known physics and chemistry. This focus on artifacts, and hence on finding evidence for extraterrestrial intelligence that is “technological” in roughly the way we are, but presumably more advanced, aligns the Drake Equation with its inspiration, the Fermi Paradox, but distances it from the primary concerns of exobiology. The relevance or even the meaningfulness of the terms appearing in the Drake equation have all been criticized; for a large sample of such criticisms, see [94]. For an alternative to the Drake Equation that estimates abiogenesis events instead of extraterrestrial intelligent systems, see [95].

The FEP suggests that the number of extraterrestrial intelligent systems is very large, but says nothing about their detectability. By characterizing all physical systems as Bayesian agents, moreover, it erases any firm distinction between “artifacts” and “products of nature”; any information-bearing pattern – anything distinguishable from noise – could be a “signal” s from a system with some level of intelligence. It suggests, therefore, that the actually important number is the conditional probability P(r|s) that a extraterrestrial signal s, whatever its form, is recognized as such, via Bayesian satisficing, by us. In this it roughly agrees with Gertz [94], who argues that the only important number is $f_{d}$, the probability of detecting intelligent extraterrestrial life by any means.

Humans are constantly bathed in large-scale information-bearing patterns, from the non-uniform distribution of visible stars to anisotropies in the cosmic microwave background [96], that we regard as “natural” and hence of no interest from a biological or cognitive-science perspective. Viewing nonrandomness at the Solar System scale as communicative similarly fails the test of utility discussed above. What, then, would be the utility of regarding a piece of space junk of clearly non-human manufacture as a communication, or as biologically meaningful? On the one hand, such a discovery would shatter our anthrocentrism, making it clear that we are not “alone in the universe” as many either fear or hope. On the other, it would confirm our anthropocentrism, by confirming our deep assumption that any beings out there are “like us” in significant ways. The utility of regarding such a discovery as not “natural”, in other words, lies not in how it allows us to better deal with our environment, but in what it tells us about ourselves. It would tell us that we are not unique, but rather in some sense inevitable. The Drake equation assumes, by including the term $f_{c}$, these poles of uniqueness or inevitability. The FEP, in contrast, challenges us to imagine a universe full of intelligent systems, the vast majority of which are nothing at all like us. It re-interprets Gertz’s $f_{d}$ as the probability that humans become smart enough to recognize intelligent extraterrestrial life when it presents itself.

A more radical take on the FEP is that it sets N=1, by definition, for any observer, whether an individual human, all of humanity, the lineage of LUCA, Gaia, or any other system, large or small. The one other intelligent system that is detectable is the detecting system’s total environment. Any system’s challenge is to understand what its total environment is trying to tell it. One thing that its total environment cannot tell it is the origin of the boundary B between them, since this event is the OOL for both the system and its total environment.

## 7. Conclusions

The OOL problem has been framed against a background of classical physics operating in classical spacetime, and assumes an abiotic environment that operates as a classical mechanism. The Conway-Kochen theorem and the Free Energy Principle challenge these assumptions, the former by showing that local determinism conflicts with known physics, and the latter by providing a formal interpretation of even classical dynamical systems as Bayesian agents. Taken together, the CK theorem and the FEP characterize physical systems as at least minimally agentive at every scale, and suggest that life, cognition, and complexity are both inseparable and ubiquitous. If this is the case, the OOL problem is replaced, in practice, with the problem of recognizing and successfully interacting with diverse intelligences in diverse embodiments, which may, but may not, involve familiar biochemistry.

## Data Availability

No new data were generated in this work.
